# Synergistic effects of berberine and adipose stem cells in a silk fibroin-PEG hydrogel for diabetic wound healing via macrophage polarization modulation

**DOI:** 10.1093/rb/rbag151

**Published:** 2026-07-21

**Authors:** Xiaohao Hu, Jia Xu, Yanping Zhong, Weihao Zheng, Chonglin Jiang, Yuer Zuo, Ke Ma, Dacai Qu, Jun Li, Sheng Xu, Li Zheng, Jinming Zhao, Hongmian Li

**Affiliations:** Department of Plastic and Aesthetic Surgery, The People’s Hospital of Guangxi Zhuang Autonomous Region & Research Center of Medical Sciences, Guangxi Academy of Medical Sciences, Nanning 530021, China; Guangxi Engineering Center in Biomedical Materials for Tissue and Organ Regeneration, The First Affiliated Hospital of Guangxi Medical University, Guangxi Medical University, Nanning 530021, China; Collaborative Innovation Centre of Regenerative Medicine and Medical Bioresource Development and Application Co-constructed by the Province and Ministry, The First Affiliated Hospital of Guangxi Medical University, Guangxi Medical University, Nanning 530021, China; Life Sciences Institute, Guangxi Medical University, Nanning 530021, China; Guangxi Engineering Center in Biomedical Materials for Tissue and Organ Regeneration, The First Affiliated Hospital of Guangxi Medical University, Guangxi Medical University, Nanning 530021, China; Collaborative Innovation Centre of Regenerative Medicine and Medical Bioresource Development and Application Co-constructed by the Province and Ministry, The First Affiliated Hospital of Guangxi Medical University, Guangxi Medical University, Nanning 530021, China; Guangxi Engineering Center in Biomedical Materials for Tissue and Organ Regeneration, The First Affiliated Hospital of Guangxi Medical University, Guangxi Medical University, Nanning 530021, China; Guangxi Engineering Center in Biomedical Materials for Tissue and Organ Regeneration, The First Affiliated Hospital of Guangxi Medical University, Guangxi Medical University, Nanning 530021, China; Collaborative Innovation Centre of Regenerative Medicine and Medical Bioresource Development and Application Co-constructed by the Province and Ministry, The First Affiliated Hospital of Guangxi Medical University, Guangxi Medical University, Nanning 530021, China; Guangxi Engineering Center in Biomedical Materials for Tissue and Organ Regeneration, The First Affiliated Hospital of Guangxi Medical University, Guangxi Medical University, Nanning 530021, China; Collaborative Innovation Centre of Regenerative Medicine and Medical Bioresource Development and Application Co-constructed by the Province and Ministry, The First Affiliated Hospital of Guangxi Medical University, Guangxi Medical University, Nanning 530021, China; Department of Plastic and Aesthetic Surgery, The People’s Hospital of Guangxi Zhuang Autonomous Region & Research Center of Medical Sciences, Guangxi Academy of Medical Sciences, Nanning 530021, China; Department of Plastic & Cosmetic Surgery, The First Affiliated Hospital of Guangxi Medical University, Guangxi Medical University, Nanning 530021, China; College of Agriculture, Guangxi University, Nanning 530004, China; College of Agriculture, Guangxi University, Nanning 530004, China; Life Sciences Institute, Guangxi Medical University, Nanning 530021, China; Guangxi Engineering Center in Biomedical Materials for Tissue and Organ Regeneration, The First Affiliated Hospital of Guangxi Medical University, Guangxi Medical University, Nanning 530021, China; Collaborative Innovation Centre of Regenerative Medicine and Medical Bioresource Development and Application Co-constructed by the Province and Ministry, The First Affiliated Hospital of Guangxi Medical University, Guangxi Medical University, Nanning 530021, China; Life Sciences Institute, Guangxi Medical University, Nanning 530021, China; Guangxi Engineering Center in Biomedical Materials for Tissue and Organ Regeneration, The First Affiliated Hospital of Guangxi Medical University, Guangxi Medical University, Nanning 530021, China; Collaborative Innovation Centre of Regenerative Medicine and Medical Bioresource Development and Application Co-constructed by the Province and Ministry, The First Affiliated Hospital of Guangxi Medical University, Guangxi Medical University, Nanning 530021, China; Guangxi Engineering Center in Biomedical Materials for Tissue and Organ Regeneration, The First Affiliated Hospital of Guangxi Medical University, Guangxi Medical University, Nanning 530021, China; Collaborative Innovation Centre of Regenerative Medicine and Medical Bioresource Development and Application Co-constructed by the Province and Ministry, The First Affiliated Hospital of Guangxi Medical University, Guangxi Medical University, Nanning 530021, China; Department of Plastic and Aesthetic Surgery, The People’s Hospital of Guangxi Zhuang Autonomous Region & Research Center of Medical Sciences, Guangxi Academy of Medical Sciences, Nanning 530021, China

**Keywords:** diabetic wound healing, adipose-derived stem cells, silk fibroin-polyethylene glycol hydrogel, berberine, macrophage polarization

## Abstract

Diabetic wound repair remains a considerable clinical challenge, largely due to the limited efficacy of current therapies. Herein, we engineered a novel silk fibroin (SF)–polyethylene glycol (PEG) hydrogel (SPB) incorporated with berberine (BBR) and adipose-derived stem cells (ASCs) to synergistically promote diabetic wound healing. By optimizing the physicochemical properties of SPB, we found that a 7:3 SF:PEG ratio provided an ideal balance of pore size (25.83 ± 3.94 μm), mechanical strength (32.33 ± 4.07 kPa) and sustained BBR release. *In vitro*, SPB significantly enhanced ASC paracrine function, promoting the production of vascular endothelial growth factor (VEGF), fibroblast growth factor 2 (FGF-2), stromal cell-derived factor 1 (SDF-1), platelet-derived growth factor-BB (PDGF-BB), and transforming growth factor beta (TGF-β). Accordingly, SF-based hydrogel system (SPBA) markedly stimulated fibroblast proliferation and migration and enhanced endothelial cell angiogenesis. Moreover, SPBA effectively modulated macrophage repolarization from the pro-inflammatory M1 to the anti-inflammatory M2 phenotype, thereby suppressing inflammation. *In vivo*, SPBA demonstrated remarkable therapeutic efficacy in diabetic rat full-thickness skin wounds, accelerating wound closure, promoting re-epithelialization, collagen deposition and neovascularization, while reducing local inflammation. These results highlight SPBA as a highly efficient and promising biomaterial strategy for diabetic wound treatment.

## Graphical abstract

**Graphical Abstract:**
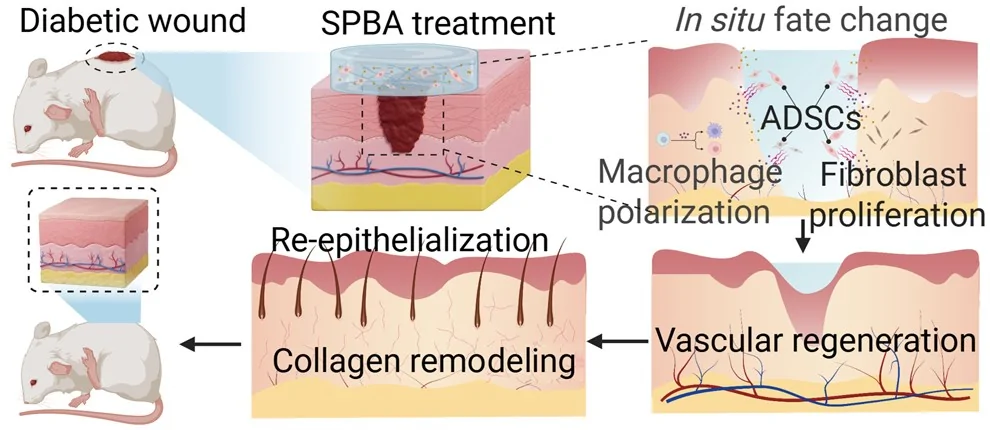

## Introduction

Impaired wound healing is a prevalent and complex complication in diabetic patients, significantly reducing their quality of life and potentially resulting in severe infections or even limb amputation [1]. The intractability of the diabetic wounds largely stems from their intricate pathophysiological underpinnings. Hyperglycemia induces microvascular dysfunction, exaggerated inflammatory responses and dysregulated immune cell activity, which collectively contribute to delayed wound healing. Moreover, impaired immune function in diabetic individuals increases susceptibility to infection, thereby compounding the challenges associated with wound management [2]. Although current therapeutic strategies—including pharmacological agents, negative pressure wound therapy and surgical procedures—offer some improvements in wound healing, they remain limited by suboptimal efficacy, high costs and adverse effects [3]. Considering the high prevalence, refractory nature and significant healthcare burden of diabetic wounds, an imperative exists for developing innovative and more effective therapeutic interventions.

Paracrine signaling is a crucial mechanism through which stem cells exert their therapeutic effects. Adipose-derived stem cells (ASCs) have demonstrated considerable promise in the field of tissue regeneration and regenerative medicine [4]. Compared to stem cells from other sources, ASCs offer distinct advantages, including ease of isolation, abundant availability, high yield, rapid proliferation and resistance to senescence [5]. Stem cells possess the capacity for multipotent differentiation into diverse cell lineages, such as vascular endothelial cells, fibroblasts and epithelial cells, thereby directly participating in the repair and reconstruction of wound tissue [6]. More importantly, ASCs can promote diabetic wound healing either through differentiation or by secreting various growth factors (e.g. PDGF, VEGF, TGF, and EGF) [7]. However, traditional ASC-based therapeutic approaches typically rely on direct cell injections, a method with significant limitations [8]. ASCs injected into the body often face challenges in maintaining long-term survival and activity at the wound site due to inadequate microenvironmental support [9]. Moreover, the complex *in vivo* pathological environment, including hyperglycemia and oxidative stress, can further impair ASC functionality [10, 11]. Crucially, deficiencies in ASC paracrine signaling significantly limit their clinical efficacy [12]. Therefore, utilizing scaffolds to provide an appropriate growth environment for ASCs, thereby enhancing their survival and paracrine function, represents an effective strategy to improve therapeutic efficacy in diabetic wound treatment [9]. Among these, hydrogels are considered ideal cell carriers due to their beneficial properties, such as a favorable 3D structure, extracellular matrix (ECM)-mimicking characteristics and high water content [13–15]. However, research on hydrogels specifically designed to enhance ASC paracrine signaling for diabetic wound treatment remains limited. Consequently, the development of hydrogels that upregulate ASC paracrine factors is crucial for advancing diabetic wound therapy.

Silk fibroin (SF), owing to its superior biocompatibility, favorable mechanical attributes and facile modification and biodegradability, is frequently used in the fabrication of hydrogels as a cell carrier, preserving cell viability and protecting cells from mechanical damage [16–19]. Studies have shown that SF-based biomaterials loaded with ASCs, which secrete various cytokines through paracrine mechanisms, exhibit promising therapeutic effects in the treatment of pelvic organ prolapse [20]. Notably, the porosity and pore size of hydrogels influence cell paracrine activity, adhesion, proliferation and migration, which are crucial for maintaining cellular homeostasis and enabling therapeutic function [18, 21, 22]. Existing research on SF hydrogels loaded with ASCs for diabetic wound treatment often overlooks the impact of hydrogel pore size on ASC behavior. Pore size regulates tissue regeneration and remodeling by influencing cell attachment, distribution and ECM secretion [18]. Therefore, developing an SF-based hydrogel with a pore size suitable for ASC growth is necessary to enhance ASC paracrine function and accelerate diabetic wound repair. Investigations into the modulation of self-assembled SF composite hydrogels have shown that polyethylene glycol (PEG) promotes the rapid and ordered stacking of SF β-sheet structures, which has the capacity to enhance the physicochemical characteristics of hydrogels [23]. However, current research on the effects of PEG on the properties of SF-based materials (such as mechanical properties, pore size and cell adhesion) is insufficient, necessitating further exploration.

Chronic diabetic wounds experience impaired healing due to a persistent inflammatory microenvironment, where excessive inflammatory factors (e.g. TNF-α, IL-6) damage the survival and function of ASCs and compromise their paracrine activity, severely reducing tissue regenerative potential [24, 25]. Berberine (BBR), an alkaloid originating from *Coptis chinensis* and other Berberis species, has attracted significant attention due to its diverse biological properties [26]. Studies suggest that BBR exhibits a dual anti-inflammatory mechanism: it inhibits the NF-κB pathway to downregulate inflammation-promoting mediators including TNF-α and IL-1β, and activates the Nrf2 pathway to enhance antioxidant defense, demonstrating efficacy in diseases like rheumatoid arthritis [27]. Concurrently, BBR directly promotes wound repair by stimulating collagen deposition and VEGF-mediated angiogenesis [28–31]. It also modulates macrophage polarization (M1→M2) and fibroblast paracrine function, stimulating the secretion of the anti-inflammatory cytokine IL-10, thus remodeling the pro-healing microenvironment [30–32]. However, poor water solubility, rapid *in vivo* metabolism and insufficient local retention result in low bioavailability of BBR, limiting its sustained action in chronic wounds [33]. Moreover, the regulation of ASC paracrine function by BBR remains underexplored.

**Figure 1:**
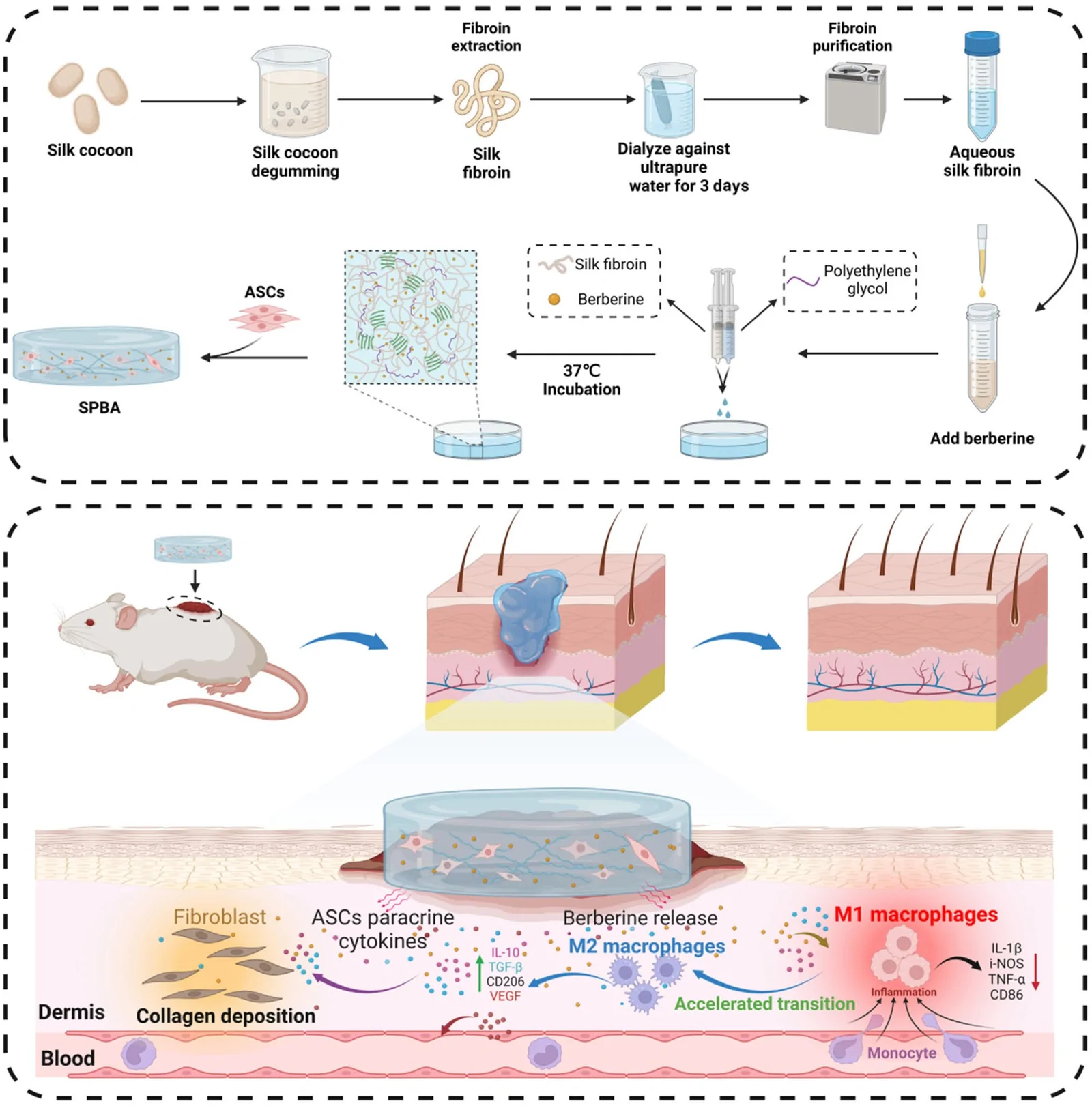
SPB hydrogel loaded with ASCs promotes the regeneration and restoration of diabetic skin ulcers through slow release of BBR and enhancement of ASCs’ paracrine function.

This study involved the fabrication of a self-assembled SPB hydrogel formulated with SF and PEG and subsequently used it to load BBR and ASCs for diabetic wound treatment. To create a 3D environment conducive to ASC growth, the present work explored the effect of SF–PEG hydrogels with varying proportions on the proliferative activity of ASCs. Leveraging its excellent anti-inflammatory properties, BBR not only alleviated local chronic inflammation in the wound but also promoted ASCs to secrete various pro-repair factors, thereby enhancing their paracrine function. This composite hydrogel promotes macrophage polarization in diabetic wounds from pro-inflammatory M1 to anti-inflammatory M2 phenotypes, accelerating wound closure and improving the quality of newly formed tissue (Figure 1). This study introduces an innovative biomaterial approach to diabetic wound treatment, showing significant potential for clinical application.

## Materials and methods

### Preparation of SPB hydrogel

An appropriate amount of silkworm cocoons was cut into fragments and subjected to boiling in a 0.02 M Na_2_CO_3_ (Aladdin, China) solution to remove sericin. This process was repeated twice, 30 min each time, followed by thorough drying in a 50°C oven. Subsequently, the desiccated SF was solubilized in a 9.3 M lithium bromide solution maintained at 60°C with thorough stirring for 6 h. The SF solution underwent dialysis against deionized water for 3 days, within a dialysis membrane (Solarbio, Beijing; molecular weight exclusion limit 8000–14000 Da), with the deionized water changed three times daily during the dialysis process. After dialysis, the SF solution was centrifuged at 4000 rpm for 10 min at 4°C to remove impurities formed during dialysis. A small portion of the SF solution was thoroughly dried and weighed to determine the concentration of the dialyzed SF solution. The concentrated SF solution was then stored at 0–4°C for later use.

At room temperature, PEG (MW 600 g/mol, MACKLIN, Shanghai) was dissolved in deionized water to form an 80% (vol/vol) solution. The concentration of the SF solution was set to 10% (wt/vol), and BBR (MACKLIN, Shanghai) was incorporated into the solution and mixed completely. The BBR-containing SF solution was then mixed with the PEG solution at ratios of 6:4, 7:3, 8:2 and 9:1 and allowed to gel by standing at room temperature in molds. Both the SF and PEG solutions were sterilized prior to mixing for subsequent cell culture and *in vivo* studies.

For the ASC-containing groups, SPA (SF-PEG hydrogel loaded with ASCs) and SPBA (SF-PEG hydrogel loaded with BBR and ASCs), ASCs were seeded onto the surface of the pre-formed hydrogel scaffolds in six-well plates. Initially, sterile SP (SF-PEG hydrogel) and SPB (SF-PEG hydrogel incorporating BBR) hydrogels were cast into the wells and allowed to crosslink completely. To ensure a stable microenvironment for cellular attachment, the scaffolds were equilibrated with complete Dulbecco’s Modified Eagle Medium (DMEM) in a humidified incubator for 12 h prior to seeding. Simultaneously, ASCs at the third to fifth passage were harvested at 80–90% confluence using 0.25% Trypsin-EDTA and collected by centrifugation at 1000 rpm for 5 min. The resulting cell pellet was resuspended in complete DMEM and adjusted to a concentration of 4 × 10^5^ cells/mL, with cell viability confirmed to be >95% by Trypan blue exclusion. Subsequently, 500 μL of the cell suspension (yielding a total of 2 × 10^5^ cells per well) was seeded dropwise onto the surface of each hydrogel scaffold. The plates were then maintained in an incubator at 37°C with 5% CO_2_ for 4 h to facilitate initial cellular anchoring and stabilization. Following the attachment period, 2.5 mL of complete DMEM was gently added to each well to provide sufficient nutrients for subsequent culture. For clarity, the experimental groups in this study are defined as follows: PBS (blank control), SP (SF–PEG hydrogel), SPB (SF–PEG + BBR), SPA (SF–PEG + ASCs) and SPBA (SF–PEG + BBR + ASCs).

### Characterization of SPB hydrogel

#### Microstructure of the SPB hydrogel

The pore structure and surface characteristics of the hydrogels were examined via scanning electron microscopy (SEM, VEGA3, Brno, Czech Republic). SPB samples prepared in 24-well culture plates were subjected to three rinses with deionized water for the elimination of excess PEG from the surface. The lyophilized samples subsequently underwent sputter-coating with a gold layer, and images were captured using SEM. Pore sizes were measured using ImageJ software.

#### Fourier transform infrared spectroscopy

The secondary structure of SPB hydrogels with different mixing ratios and pure SF protein was examined by Fourier transform infrared spectroscopy (FTIR; Bruker Vertex 33, Germany). For sample preparation, lyophilized hydrogel samples and pure SF protein were ground with spectroscopic-grade potassium bromide and pressed into pellets. Spectra were acquired in the range of 400–4000 cm^−1^ with a spectral resolution of 2 cm^−1^. An accumulation of 32 scans was performed to ensure a high signal-to-noise ratio.

#### Compression test

The mechanical attributes of hydrogels were measured using a universal material testing system (50 N; Instron 5943, USA). A compressive force was applied with a displacement velocity of 4.8 mm/min until the point where the hydrogel, measuring 10 mm in diameter and 10 mm in height, fractured. The elastic modulus in the elastic deformation region, defined as the gradient of the linear elastic region within the stress-strain plot, was calculated and derived from the stress–strain curve.

#### Adhesion testing

Lap-shear tensile strength measurements were performed using a universal testing machine (50 N; Instron 5943, USA). Briefly, fresh porcine skin was cut into rectangular sections of 25 mm × 50 mm and fixed onto two glass slides using cyanoacrylate adhesive. The pre-formed SPB hydrogels were placed between the overlapping porcine skin substrates to establish a defined bonding area of 25 mm × 25 mm. To ensure tight interfacial contact and stable bonding, each specimen was compressed under a 100-g load and held for 30 min prior to testing. Tensile loading was applied at a steady crosshead speed of 10 mm/min until the complete debonding of the tissue–hydrogel interface, and the maximum shear stress was recorded as the lap shear strength. The shear strength was calculated by dividing the maximum load by the overlapping bonding area. Each experimental group contained at least three independent replicates.

#### Swelling behavior

The hydrogels’ swelling capacity was ascertained to measure their water absorption capacity. Hydrogels were dried for 24 h using a vacuum dryer (Beijing Sihuan Furui Keyi, LGJ-10C, China), and their initial weight was recorded as *W*_0_. Subsequently, the dried samples were submerged in phosphate-buffered saline (PBS, pH 7.4) and agitated in a shaker at 37°C (100 rpm). At predetermined time points, samples were removed, blotted with filter paper and subsequently weighed, with the weight recorded as *W_t_*. The swelling ratio was computed utilizing the following formula:


$$\text{Swelling ratio}\left(\%\right)=\;(W_{t}-W_{0})/W_{0}\times \text{ 1}00\%.$$

#### *In vitro* degradation

The *in vitro* degradation behaviors of the hydrogels were characterized by monitoring their gravimetric mass loss over a 21-day period. Briefly, pre-formed hydrogel samples from each group were initially freeze-dried and weighed to record their baseline dry weight (*W*_0_). The lyophilized specimens were then individually immersed in sealed vials containing 5 mL of PBS (pH 7.4) and maintained in a thermostatic shaking incubator at 37°C with a continuous agitation speed of 50 rpm. At predetermined incubation intervals (1, 3, 5, 7, 10, 14 and 21 days), the corresponding hydrogel samples were retrieved from the buffer. To ensure accurate gravimetric measurement, the collected residues were gently rinsed with deionized water to remove residual salts from the PBS, followed by a secondary lyophilization process. The final dry weight of the degraded hydrogel at each time point was recorded as *W_t_*. The degradation ratio, representing the cumulative mass loss percentage, was calculated according to the following equation:


$$\text{Degradation ratio }\left(\%\right)\;=\;\left(W_{0}-W_{t}\right)/W_{0}\times \text{ 1}00\%.$$

All measurements were performed in triplicate (*n* = 3) for each formulation at each designated time point to ensure reproducibility.

#### X-ray diffraction (XRD)

The crystalline structures of the lyophilized pure SF, pure PEG and composite SPB hydrogels with various formulations were investigated using an X-ray diffractometer (Bruker, Karlsruhe, Germany) equipped with a Cu-Kα radiation source. The measurements were conducted at an operating voltage of 40 kV and a current of 40 mA. Diffractograms were recorded over a 2θ range of 3° to 90° with a step size of 0.1° and a scanning speed of 2°/min.

#### Drug release and quantification

Hydrogels were enclosed within dialysis bags and subsequently submerged in 50 mL of PBS buffer solution. The setup was agitated in a shaker at a constant rate of 50 rpm at 37°C. At predetermined time points, 3 mL of the supernatant was aliquoted for analysis. The optical density of the solution was quantified at a wavelength of 340 nm utilizing a UV-Vis spectrophotometer (Puxi, Beijing, China).

### SPB evaluation of the biological effect of SPB hydrogel *in vitro*

#### Cell proliferation assay

SPB hydrogels were incubated in 2 mL of high-glucose DMEM medium supplemented with 10% fetal bovine serum (FBS) and 1% penicillin/streptomycin at 37°C for 5 days. The resulting release supernatant from the SPB (containing an optimized concentration of 200 mg/L BBR, as determined by preliminary CCK-8 assays in Supplementary Figure S1) was harvested and sterile-filtered. According to the manufacturer’s instructions, the effect of the SPB release buffer on the proliferation of rat ASCs, and rat fibroblasts was assessed using a CCK-8 kit (Biosharp, China). Cells (1 × 10^4^) were seeded into individual wells of 96-well plates (Biosharp, China) and maintained in complete culture medium (high-glucose DMEM supplemented with 10% FBS and 1% penicillin/streptomycin) for 24 h. Subsequently, the supernatant was replaced with the SPB release buffer for further culture. DMEM supplemented with 10% FBS and 1% penicillin/streptomycin served as the control.

#### Biocompatibility assessment of SPB hydrogel

To determine the effect of SPB hydrogel on cell viability and morphology, this experiment analyzed the spreading area of ASCs within the SPB hydrogel. Cell viability was assessed via Live/Dead staining assay (Solarbio, China) and imaged with a confocal microscope.

#### ELISA

To determine the effect of SPB on the paracrine function of ASCs, ASCs were seeded onto regular cell culture dishes, SP hydrogels and SPB hydrogels, respectively. After 12 h of culture in serum-free medium, the cell culture supernatants were collected from each group and subsequently subjected to enzyme-linked immunosorbent assay (ELISA) to detect the content of various cytokines.

#### Tube formation assay

The effect of SPB hydrogel loaded with ASCs (SPBA) on the tube formation of human umbilical vein endothelial cells (HUVECs) underwent assessment through a tube formation assay. Briefly, 50 μL of Matrigel (ABW, China) was seeded into individual wells of a 96-well plate and allowed to polymerize by incubating at 37°C with 5% CO_2_ for 30 min. HUVECs were diluted with SPBA release buffer, and 100 μL of cell suspension (2 × 10^5^ cells/mL) was then seeded into each well and cultured for 6 h. Finally, capillary-like structures were photographed using a microscope (Olympus, Tokyo, Japan). High-glucose DMEM medium without serum served as the control. Results were analyzed using ImageJ.

#### Cell migration assay

The impact of SPBA on fibroblast migration capacity was assessed via a cell migration assay. Fibroblasts were cultured in six-well plates. When cells reached 80% confluence, a confluent cell monolayer was gently scratched across the entire diameter of the culture dish using a pipette tip. Subsequently, cells underwent rinsing with culture medium to eliminate all detached cells, followed by treatment with different conditioned media (as previously described). Images were captured at two time points, 0 and 24 h, using an inverted fluorescence microscope (Echo Revolve, USA). The area of the scratch wound was measured using ImageJ software.

#### Macrophage polarization

Bone marrow-derived macrophages were harvested from C57BL/6 mice. Cells were cultured in complete medium (DMEM supplemented with 10% FBS, 1% penicillin/streptomycin and 30 ng/mL M-CSF) supplemented with 100 ng/mL lipopolysaccharide (LPS) and 20 ng/mL interferon-gamma (IFN-γ) for a 24-h period to induce M1 polarization. The following experiments were then performed: (1) flow cytometry: the percentages of CD86^+^ (pro-inflammatory) and CD206^+^ (anti-inflammatory) macrophages were determined by flow cytometry after different treatments; (2) reverse transcription-quantitative polymerase chain reaction (RT-qPCR): total RNA was extracted from macrophages using Trizol reagent. First-strand cDNA was synthesized using the Advantage® RT-for-PCR Kit (Takara, China). Real-time quantitative PCR was conducted utilizing Genious 2X SYBR Green Fast qPCR Mix (ABclonal, China). GAPDH was used as the endogenous control gene, and primer sequences are provided in a separate Table 3. For immunofluorescence, cells were immobilized with 4% paraformaldehyde at ambient temperature for 10 min, then subjected to three washes with PBS-T. Blocking was carried out using PBS-T supplemented with 1% bovine serum albumin at ambient temperature for 1 h. Primary antibodies, diluted 200-fold, were added to the blocking solution and incubated overnight at 4°C. After three washes with PBS-T, secondary fluorescent antibodies were incubated at room temperature in the dark. Subsequently, cells were washed three more times with PBS-T, and nuclei were counterstained with DAPI before observation under an inverted fluorescence microscope (Echo Revolve, USA).

### Animal experiments

This animal study was approved by the Animal Ethics Committee of Guangxi Medical University and conducted (NO: 202211109) according to the guidelines of the National Health and Medical Research Council (China). Male SD rats (180–200 g) were selected, and diabetes was induced by intraperitoneal injection of streptozotocin solution (60 mg/kg). Rats were classified as diabetic once their blood glucose concentrations surpassed 300 mg/dL. After anesthetizing the rats with isoflurane, the dorsal hair was removed using a shaver and depilatory cream. Subsequently, two full-thickness circular skin wounds, each 1.5 cm in diameter, were created on both sides of the dorsal midline of each rat. Wounds were treated with different methods (PBS, SP, SPB, SPA, SPBA). Throughout the entire experimental process, all procedures, such as wound creation and protective dressing application, were kept consistent across groups, except for the specific interventions, to eliminate environmental variations. Wound photographs were taken regularly, and tissue specimens were collected. The rate of healing, quantified as a percentage of the initial surgical wound area, was determined by quantifying the recovered wound area using ImageJ software (National Institutes of Health, Bethesda, MD, USA).

### Histological analysis

Rats were euthanized via intraperitoneal administration of an excessive dose of 0.6% sodium pentobarbital. Wound tissues were collected on post-operative days 7 and 21, then fixed in 4% paraformaldehyde, and subsequently paraffin-embedded following dehydration. The paraffin-embedded tissues were then sectioned into 5-μm-thick slices. These sections were then stained with hematoxylin and eosin (H&E) and Masson’s trichrome, respectively, to observe the length of newly formed epithelium and collagen maturity.

### Immunohistochemistry staining analysis

The deparaffinization and rehydration steps for the sections were identical to those described for H&E staining. Antigen retrieval was performed following the instructions provided in the kit. Endogenous peroxidase was blocked using hydrogen peroxide. Subsequently, sections underwent three washes with PBS buffer. Samples were incubated with primary antibodies, followed by three more washes with PBS buffer. An enhanced enzyme-labeled goat anti-mouse IgG polymer was incubated on the tissue sections at 37°C, followed by DAB chromogenic development. Nuclear counterstaining was performed with hematoxylin. Tissue sections were observed under an inverted fluorescence microscope (Echo Revolve, USA). Images were processed and analyzed using ImageJ software.

### qPCR analysis

Total RNA was isolated from wound tissues employing Trizol (Invitrogen). Total RNA (1 μg) was reverse-transcribed into cDNA using the PrimeScript™ RT reagent kit with gDNA Eraser (RR 047 A, TaKaRa, Otsu, Shiga, Japan). qPCR was performed using TB Green® Premix Ex Taq™ (Tli RNaseH Plus) (RR 820 A, TaKaRa, Otsu, Shiga, Japan). PCR was conducted on a CFX 96™ optical reaction module (Bio-Rad, Hercules, CA, USA). The primer sequences employed for qPCR are provided in Table S1.

### Statistical analysis

All quantitative data are presented as mean ± standard deviation (SD). Statistical analyses were performed using GraphPad Prism 9.0 (GraphPad Software, San Diego, CA, USA). For comparisons involving more than two groups, one-way analysis of variance was employed. All experiments were performed with at least three independent biological replicates.

## Results and discussion

### Characterization of SPB hydrogel

**Figure 2:**
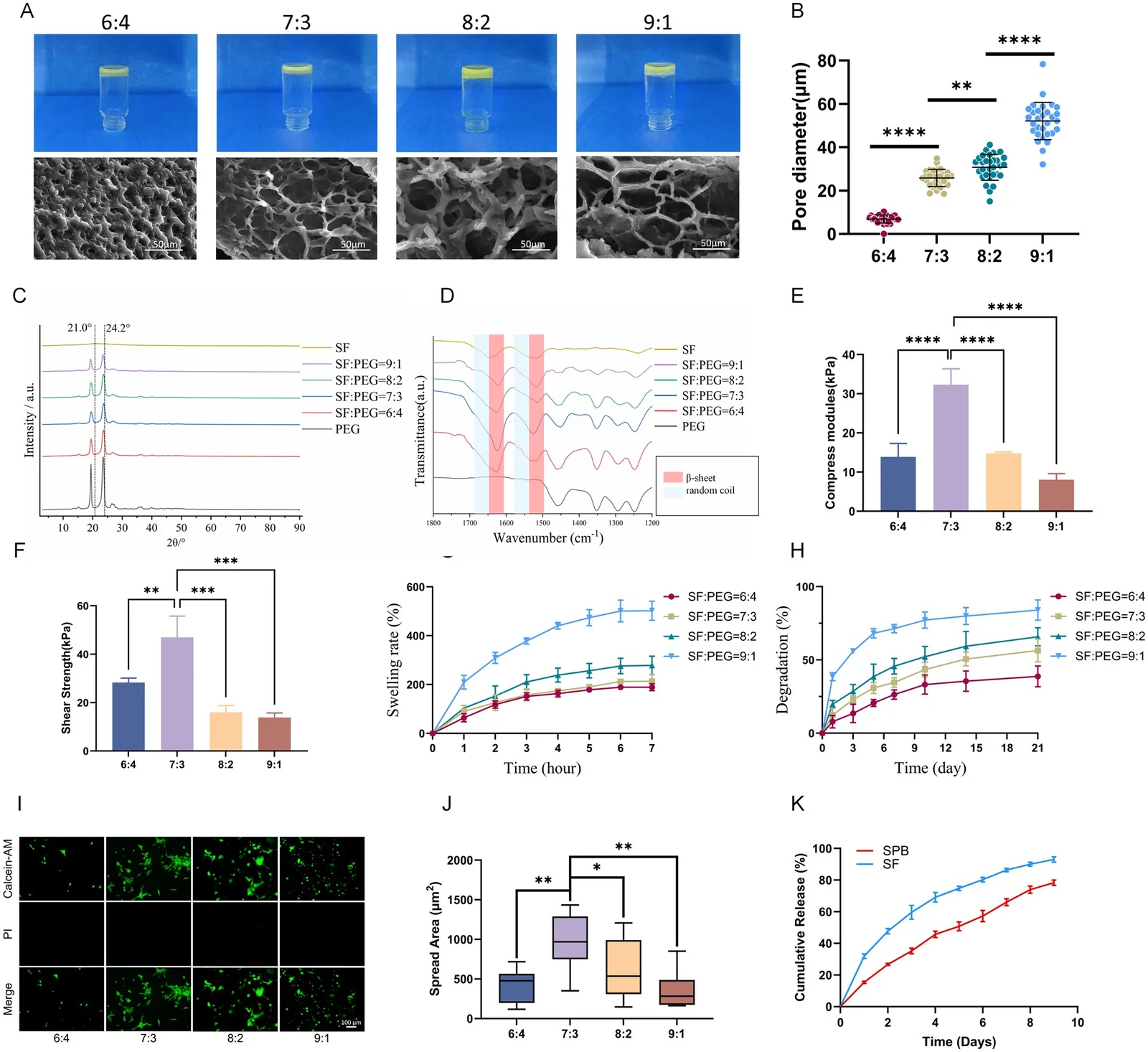
Material properties of SPB hydrogel. (**A**) Gelation of SPB hydrogels and their corresponding morphology under scanning electron microscope (scale bars: 50 μm). (**B**) Detection of pore size of SPB hydrogel by scanning electron microscope (*n* = 30). (**C**) XRD analysis of the SPB hydrogel. (**D**) FTIR spectrum of SPB hydrogels. (**E**) Compressive modulus analysis of SPB hydrogel by stress–strain experiment. (**F**) Lap-shear strength of SPB hydrogels with varying SF:PEG ratios (*n* = 3). (**G**) Swelling kinetics of the hydrogel formulated with varying proportions of PEG (*n* = 3). (**H**) *In vitro* degradation behavior of the hydrogels as determined by cumulative weight loss percentage over a 21-day incubation period in PBS at 37°C (*n* = 3). (**I**) Fluorescent micrographs on Day 1 depicting the morphology of ASCs seeded onto SPB hydrogels with different SF–PEG ratios (scale bars: 100 μm). (**J**) Box-whisker distribution graphs illustrating the spread area for ASCs on SPB hydrogels. (*n* = 10). (**K**) Drug release speed of SF hydrogel and SP hydrogel (*n* = 3) (*P < 0.05, **P < 0.01, ***P < 0.001, ****P < 0.0001).

To prepare hydrogels conducive to ASC growth, we investigated four different SF and PEG ratios. SEM results showed that all four concentration ratios of SPB exhibited uniform and interconnected porous microstructures (Figure 2A). As the PEG concentration decreased, the pore size of SPB increased, with the average pore size rising from 6.87 ± 1.91 μm to 52.11 ± 8.59 μm (Figure 2B). Studies suggest that hydrogel pore sizes typically range from 1 to 100 μm; if the pore size is too small, cell growth is restricted, impairing specific cellular functions, while excessively large pores prevent proper cell adhesion and growth [34]. Most animal cells range from 10 to 20 μm in diameter [35]. The 7:3 SPB hydrogel exhibited a pore size of 25.83 ± 3.94 μm, indicating its potential as an ideal cell carrier.

As wound dressings, hydrogels must possess elasticity and mechanical strength appropriate for tissue adaptability [36]. To facilitate a direct comparison of the crystalline structures across different formulations, all XRD diffractograms were normalized to the SF matrix scattering intensity at 2θ = 21.0° (Figure 2C). In the normalized profiles, the dominant crystalline reflections of PEG (at 19.5° and 23.6°) exhibited a gradual decline as the PEG content decreased, which is physically consistent with the mass fraction effect. Notably, a significant “shoulder elevation” was observed at 2θ = 24.2°, a characteristic reflection of the Silk II (β-sheet) crystalline lattice. Specifically, the normalized intensity of the 7:3 SPB group at this position remained as high as 4.72 a.u., markedly surpassing the 2.88 a.u. observed in the pure PEG group. This anomalous intensity reinforcement and asymmetric peak broadening provide rigorous crystallographic evidence that the SF β-sheet diffraction constructively overlaps with the primary PEG signal, indicating that the 7:3 ratio provides the optimal molecular environment for SF crystalline self-assembly.

These crystallographic findings were further corroborated by FTIR spectroscopy at the molecular chain level (Figure 2D). SF exhibited highly conformation-sensitive absorption within the Amide I (1700–1600 cm^−1^) and Amide II (1600–1500 cm^−1^) bands. Pure PEG shows no characteristic absorption peaks in these regions, indicating its negligible influence on structural properties, and thus can serve as a basis for evaluating hydrogel structural changes [17]. At the 7:3 SF:PEG ratio, the intensities of these characteristic bands reached their maximum, with a distinct red-shift toward the frequencies representative of β-sheet structures (approximately 1625 and 1520 cm^−1^). The maximization of β-sheet absorption in FTIR aligns perfectly with the elevated 24.2° signal observed in the XRD patterns. Together, these dual characterizations demonstrate that 30% PEG acts as an efficient structural inducer, facilitating the dehydration and transition of SF macromolecular chains from random coils into highly ordered Silk II β-sheet domains [37]. This reinforced crystalline network provides the fundamental microstructural basis for the superior mechanical integrity and enhanced degradation resistance observed in the SPB hydrogel.

To assess the mechanical characteristics of the four SPB hydrogels, we measured their compressive moduli. As shown in Figure 2E, varying the PEG concentration from 40% to 10% resulted in different compressive moduli for the SPB hydrogels. The compressive modulus was highest when the PEG concentration was 30% (32.33 ± 4.07 kPa), markedly greater than that observed in all the remaining groups. This difference reached statistical significance (*P* < 0.05) and is consistent with the inference drawn from the aforementioned FTIR data. This demonstrates that the 7:3 SF:PEG ratio SPB hydrogel exhibited the highest mechanical strength.

The lap shear strength of the hydrogels was evaluated to assess their tissue-bonding capability (Figure 2F). The 7:3 ratio (SPB) achieved the highest adhesive strength at 46.93 ± 8.81 kPa, significantly outperforming the 6:4 (28.27 ± 1.85 kPa), 8:2 (16.00 ± 2.77 kPa) and 9:1 (13.87 ± 1.85 kPa) groups. This superior adhesion is attributed to the balance between cohesive integrity and interfacial binding. Specifically, the 7:3 ratio combines a robust SF β-sheet network for internal strength with sufficient PEG-mediated elasticity for tissue wetting and hydrogen bonding. In contrast, higher PEG content (6:4) compromised cohesive strength, while lower PEG levels (8:2 and 9:1) resulted in an overly rigid matrix that limited molecular contact with the tissue surface. Consequently, the 7:3 formulation provides the optimal mechanical equilibrium for stable and secure wound coverage.

The swelling ratio of hydrogels is a crucial parameter determining their ability to absorb biological fluids and maintain a moist environment [38, 39]. Results showed that as immersion time increased, the swelling ratio of all hydrogels rapidly increased, reaching maximum and equilibrium swelling within 7 h (Figure 2G). The hydrogel with a 30% PEG concentration exhibited a swelling ratio (213.9% ± 28.21%) significantly lower than those with 20% and 10% PEG concentrations (278.6% ± 37.36% and 502.1% ± 38.97%, respectively), and slightly higher than that with 40% PEG concentration (189.2% ± 27.66%). Studies have shown that the β-sheet structure in the crystalline regions exhibits strong hydrophobicity and water stability [40]. For SF hydrogel materials with higher crystallinity, the swelling ratio is generally lower. Therefore, as the addition of 30% PEG promotes the production of more β-sheets in SF, SPB at this ratio maintains good structural stability, preventing significant swelling.

Specifically, the 6:4 SF:PEG hydrogel, possessing the smallest pore size (6.87 ± 1.91 μm) and highest PEG content, exhibited the lowest swelling ratio (189.2 ± 27.66%). This is likely due to the combined effect of a denser network structure (implied by smaller pores) and the high concentration of hydrophilic PEG, which, despite its hydrophilicity, might contribute to a more compact network that limits overall water uptake capacity under these specific conditions [41]. The 7:3 SF:PEG hydrogel, while having a significantly larger pore size (25.83 ± 3.94 μm), showed a swelling ratio (213.9% ± 28.21%) only slightly higher than the 6:4 group, without a proportionally large increase. This can be primarily attributed to its highest β-sheet content, which imparts greater structural stability and hydrophobicity to the SF component, thereby counteracting the tendency for increased swelling that might otherwise be expected from its larger pore size. Conversely, the 8:2 and 9:1 SF:PEG groups exhibited higher swelling ratios. This is because, with further reduction in PEG content, both the β-sheet content decreases (leading to lower crystallinity and stability) and the pore size increases significantly, resulting in a looser, less stable network with reduced hydrophobic interactions, thus allowing for greater water absorption [42].

The *in vitro* degradation profiles of the SPB hydrogels over 21 days revealed a composition-dependent mass loss (Figure 2H). The 6:4 and 7:3 formulations exhibited comparable degradation kinetics, with cumulative mass losses of 38.82 ± 7.09% and 56.43 ± 7.81% by Day 21, respectively. In contrast, the 8:2 and 9:1 groups displayed significantly accelerated degradation rates, reaching 65.82 ± 6.18% and 84.00 ± 6.99% at the same time point. This trend indicates that higher PEG concentrations contribute to a more stable hydrogel network, whereas reduced PEG content leads to a more hydrophilic and less stable structure, resulting in faster hydrolytic erosion.

The cell-spreading area is a key indicator for evaluating the biocompatibility of hydrogels. Ideal hydrogel materials should promote effective cell-hydrogel interactions; otherwise, cells may struggle to adhere, appearing rounded and potentially undergoing anoikis [36, 43]. This study analyzed the spreading area of ASCs cultured on SPB hydrogels with four different PEG concentration ratios. As shown in Figure 2I, very few dead cells were discernible within the stained images, indicating good hydrogel biocompatibility. In hydrogels with 40 and 20% PEG concentrations, many cells exhibited a rounded morphology, suggesting poor cell adhesion. In contrast, cells on the SPB hydrogel with 30% PEG concentration appeared triangular or spindle-shaped, with extensive spreading and numerous filopodia, indicating that this PEG ratio provided excellent attachment sites for cell growth. Furthermore, we quantified the cell spreading areas. As shown in Figure 2J, the cell spreading area was largest at 30% PEG concentration (976.5 ± 340.7 μm^2^) and smallest at 10% PEG concentration (355.0 ± 225.0 μm^2^). Based on these comprehensive experimental results, the SPB hydrogel with a 30% PEG concentration was selected for further investigation.

Sustained and controlled drug release capability is essential for effective drug delivery systems. Studies have shown that Silk-PEG hydrogels are excellent sustained-release carriers, capable of continuously delivering poorly soluble minoxidil to the inner ear for treatment [44]. However, the ability of this hydrogel type to load and sustain the release of BBR has been rarely reported. To investigate the BBR release rate from the hydrogels, we established a drug release system and determined the release profiles of BBR from SPB and pure SF hydrogels. As shown in Figure 2K, SPB significantly reduced the rapid burst release of BBR. BBR loaded in SF hydrogels released approximately 60% within 3 days, whereas the SPB group released only about 35%, indicating a significantly higher release of BBR from SF hydrogels compared to SPB. After 9 days of incubation, the drug release rate from SF hydrogels reached approximately 93%, while that from SPB hydrogels was only about 78%. This observation can be explained by the fact that in SF-based sustained-release systems, drug molecules can be distributed in both amorphous, non-crystalline regions and potentially within the β-sheet layers of crystalline regions. The amorphous regions are characterized by disordered molecular chains and possess larger interstitial spaces, allowing water molecules to easily penetrate these areas, resulting in swelling of the amorphous regions [45]. Conversely, the stable structure of Silk II crystals delays drug release from these regions. Therefore, we speculate that the sustained-release effect of BBR in SPB is due to the increased proportion of crystalline regions in the hydrogel material resulting from the incorporation of PEG.

Achieving high biocompatibility stands as an essential requirement for the successful practical application of materials. To ensure the therapeutic effect of BBR and avoid potential cytotoxicity, its optimal concentration must be determined [46]. To evaluate the viability of ASCs cultured on SPB hydrogels with varying BBR concentrations, cell proliferation detection was employed. Supplementary Figure S1 illustrates that by culture day 3, ASC proliferation rates, when stimulated by SPB culture supernatants containing 100 and 200 mg/L BBR, rose by 9 and 19%, respectively, demonstrating statistically significant increases relative to the control group (*P* < 0.0001). Conversely, BBR concentrations of 400 and 800 mg/L led to cell proliferation rates that were notably diminished relative to the control group, thereby pointing to cytotoxic outcomes at these levels. Therefore, in subsequent experiments, a concentration of 200 mg/L was used.

### Paracrine function of ASCs within SPB hydrogel

To ensure the reliability of the secretome analysis and eliminate potential bias caused by uneven cell density, we first verified the spatial distribution of ASCs within the SPB hydrogel. 3D confocal laser scanning microscopy reconstruction demonstrated that the ASCs had uniformly colonized the interconnected porous architecture of the hydrogel (Supplementary Figure S2). The cells exhibited extensive spreading and high viability throughout the scaffold, confirming a homogenous cell distribution. This consistent cellular environment provides a robust biological foundation for accurately quantifying the paracrine activity of the cells.

The paracrine effect of stem cells is a core mechanism through which they exert therapeutic effects in tissue repair and regenerative medicine. Traditionally, stem cells were thought to primarily repair damage by differentiating into specific tissue cells. Nevertheless, a growing volume of evidence indicates that diverse bioactive molecules released by stem cells, including growth factors, chemokines and extracellular vesicles, exert a more pivotal influence on modulating the conduct of neighboring cells and fostering tissue regeneration and repair [12]. Therefore, it is essential to assess the impact of hydrogels on the paracrine function of stem cells.

**Figure 3:**
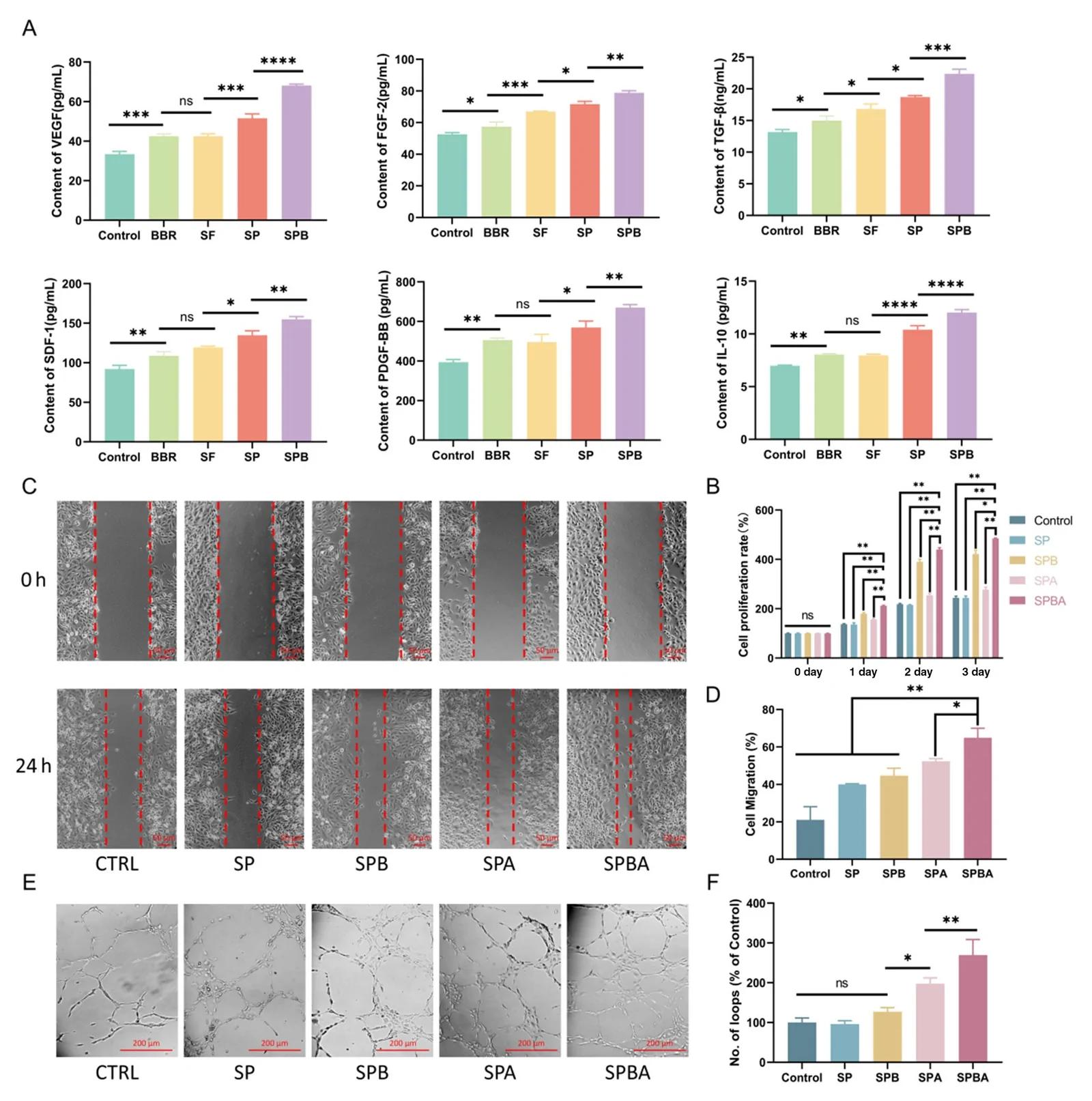
The biological effect of SPBA *in vitro*. (**A**) Paracrine function of ASCs affected by SPB using ELISA assay (*n* = 3). (**B**) Effect of SPBA on fibroblast proliferation detected via CCK-8 assay (*n* = 3). (**C**, **D**) Effect of different interventions on fibroblast migration and statistical analysis chart (scale bars: 50 μm, *n* = 3). (**E**, **F**) Tube formation by vascular endothelial cells in matrix gel following different treatments and statistical analysis chart (scale bars: 100 μm, *n* = 4) (*P < 0.05, **P < 0.01, ***P < 0.001, ****P < 0.0001, ns: the difference was not statistically significant).

The paracrine activity of ASCs within SPB hydrogels was evaluated using ELISA kits. The quantitative analysis of these cytokines was performed based on standard curves generated for each respective cytokine, as detailed in Supplementary Figure S4. The result demonstrates that ASCs cultivated within SPB hydrogels exhibited markedly elevated secretion of various cytokines, including SDF-1, TGF-β, VEGF, FGF-2, PDGF-BB and IL-10. These respective cytokine levels showed increases of 1.7-, 1.7-, 2.0-, 1.5-, 1.7- and 1.7-fold, representing statistically significant enhancements relative to the control group (*P *< 0.01, Figure 3A). Prior investigations have established that hydrogel stiffness profoundly impacts the paracrine activity of stem cells [47]. Furthermore, BBR’s capacity to modulate cellular paracrine function has been evidenced in previous studies [30, 31]. However, the specific impact of BBR on the paracrine function of stem cells, especially regarding growth factors and anti-inflammatory factors, remains underexplored. Our findings suggest that the concentrations of various cytokines were notably increased in the BBR group when contrasted with the control group. This suggests that BBR stimulates cells to release more therapeutic growth factors and anti-inflammatory factors. Furthermore, cytokine concentrations within the SP hydrogel group were notably elevated compared to the SF group, suggesting that PEG incorporation enhanced the hydrogel’s stiffness, consequently augmenting the paracrine function of ASCs. In summary, the SPB hydrogel likely promotes the paracrine secretion of various cytokines by ASCs through the combined effects of enhanced hydrogel stiffness and BBR incorporation, collectively maintaining high cellular activity.

### SPBA promotes fibroblast proliferation and migration

An optimal hydrogel dressing ought not only to furnish a moist milieu for the wound, thereby facilitating cellular physiological activities, nutrient delivery and oxygen exchange, but also to expedite wound healing via the therapeutic agents it delivers and the diverse growth factors secreted by encapsulated stem cells [13–15]. Fibroblasts are pivotal in the wound-healing process, given their responsibility for synthesizing and secreting ECM constituents, such as collagen, elastin and glycosaminoglycans, which collectively establish the structural framework for wound repair [48].

The impact of SPB hydrogel loaded with ASCs (SPBA) on fibroblast proliferation was determined through a CCK-8 cell proliferation assay. Figure 3B illustrates that, relative to the control group, the SP release buffer did not exert a significant effect on fibroblast proliferation, nor did it inhibit it. In contrast, fibroblasts cultured with conditioned media from the BBR and ASCs groups exhibited significantly enhanced proliferation rates. Previous research has shown that an appropriate concentration of BBR can promote fibroblast proliferation, with a proliferation rate approximately 1.5 times that of the control group [46]. Within this investigation, the cell proliferation rate observed in the SPBA group was 2-fold higher than that of the control group, a disparity that proved statistically significant (*P *< 0.01). This promotion of fibroblast proliferation was greater than that observed in previous studies, suggesting that SPBA combines the advantages of BBR’s sustained release and ASCs’ paracrine function, synergistically enhancing fibroblast proliferation.

Figure 3C presents the outcomes of the fibroblast scratch assay. At 24 h, fibroblast migration rates in the SPB and SPA groups were 2.1-fold (*P* < 0.001) and 2.5-fold (*P* < 0.0001) higher, respectively, when compared to the control group (Figure 3D) The SPBA group’s release buffer demonstrated a significantly stronger effect in promoting fibroblast migration than all other groups, with statistically significant differences, which was largely consistent with the CCK-8 assay results.

### *In vitro* endothelial cell angiogenesis analysis

Currently, merely a restricted number of investigations have indicated BBR’s capacity to foster angiogenesis during wound repair [49]. Research by Liu *et al*. has shown that composite microneedles carrying BBR and Baicalein in nanocarriers possess the ability to promote angiogenesis [50]. However, whether BBR alone, when incorporated into such composite microneedles, can promote angiogenesis has not been extensively investigated. Our study revealed that BBR, when used alone, exhibited a certain pro-angiogenic effect (Figure 3E and F), confirming that BBR is indeed an effective component for promoting angiogenesis. The SPBA group exhibited the most pronounced capacity to stimulate HUVEC angiogenesis, yielding a significantly greater number of new vessels than all other experimental groups, with these differences being statistically significant (*P* < 0.01). This finding demonstrates that SPBA can significantly enhance the angiogenic capacity of HUVECs.

### SPBA inhibits inflammatory response by regulating macrophage M1 and M2 polarization

Ample research has demonstrated that the failure of macrophage polarization from M1 to M2 leads to impaired diabetic wound healing and may even result in wound infection [20, 51]. Therefore, controlling inflammation and promptly shortening the inflammatory phase are crucial for breaking this vicious cycle [52]. Both ASCs and BBR have been shown to possess the ability to regulate inflammation [53, 54]. However, their specific effects and mechanisms in regulating inflammation within diabetic wounds remain underexplored. To address this, we treated LPS/IFN-γ-induced macrophages using various interventions and examined their polarization.

**Figure 4:**
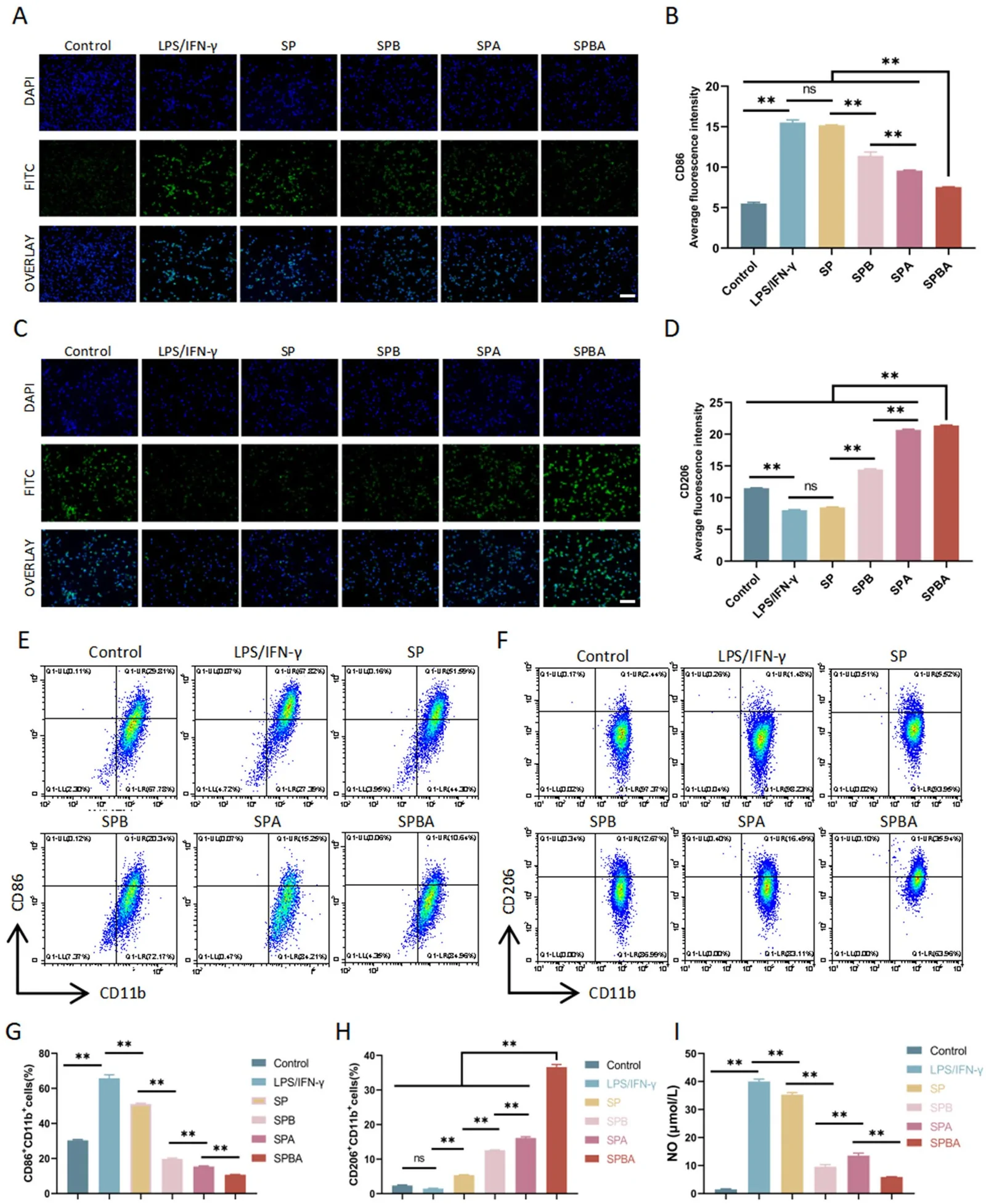
SPBA modulates the phenotype and function of macrophages. (**A**, **B**) Immunofluorescence analysis was performed to assess the expression of CD86 within macrophages subjected to different interventions induced by LPS and IFN-γ, followed by quantitative analysis of the percentage of CD86 expression (scale bars: 100 μm, *n* = 3). (**C**, **D**) Immunofluorescence analysis was performed to assess the expression of CD206 within macrophages subjected to different interventions induced by LPS and IFN-γ, followed by quantitative analysis of the percentage of CD206 expression (scale bars: 100 μm, *n* = 3). (**E**–**H**) The proportion of CD86- and CD206-positive cells in macrophages after inflammation induced by LPS and IFN-γ was detected by flow cytometry and quantitative analysis (*n* = 3). (**I**) Griess reagent was used to detect the effects of different treatments induced by LPS and IFN-γ on the expression of NO in macrophages (*n* = 3) (**P < 0.01, ns: the difference was not statistically significant).

Immunofluorescence analysis revealed that CD86 expression in macrophages from both the SPB and SPA groups was markedly diminished, decreasing to 0.73-fold and 0.62-fold, respectively, compared to the LPS/IFN-γ group (*P *< 0.01, Figure 4A and B). The downregulation of CD86 was most pronounced in the SPBA group, which was reduced to 0.49 times that of the LPS/IFN-γ group (*P* < 0.01). Conversely, CD206 expression in the SPB, SPA and SPBA groups demonstrated a substantial upregulation, rising to 1.80, 2.57 and 2.66 times that of the LPS/IFN-γ group, respectively (Figure 4C and D). These statistically significant findings (*P* < 0.01) collectively suggest that SPBA profoundly influenced the expression of both CD206 and CD86 in macrophages.

Flow cytometry was employed to quantify the cellular proportions of CD86^+^ and CD206^+^ macrophages. As depicted in Figure 4E–H and Supplementary Figure S3, SPBA significantly reduced the proportion of CD86^+^ macrophages while concurrently increasing the proportion of CD206^+^ macrophages. We infer that SPBA promotes macrophage polarization from the M1 to the M2 phenotype, exhibiting superior anti-inflammatory effects compared to ASCs or BBR used individually. This outcome is likely attributable to the synergistic effect of increased anti-inflammatory factor secretion from highly active ASCs within the hydrogel, combined with the anti-inflammatory properties of BBR.

During inflammation, nitric oxide (NO) released by M1 macrophages helps combat foreign pathogens [55]. However, excessive NO production is toxic, as it forms free radicals such as superoxide, which leads to the generation of toxic peroxynitrite, further exacerbating inflammation and organ damage [56]. To evaluate NO production by macrophages, we measured nitrite levels using the Griess assay. Figure 4I illustrates that stimulation with LPS and IFN-γ led to a substantial increase in macrophage NO levels, which were 26-fold greater than those observed in the untreated control group (*P* < 0.01). Conversely, following stimulation of macrophages with SPB and SPA treatments, a notable decrease in nitrite production was detected in the culture medium, reaching 0.24- and 0.34-fold, respectively, compared to the LPS/IFN-γ group. These statistically significant results (*P* < 0.01) collectively indicate that SPBA suppressed NO release from macrophages, thereby mitigating inflammatory progression.

### SPBA promotes healing of wounds

The therapeutic effectiveness of SPBA in promoting wound closure within living organisms was rigorously evaluated using a full-thickness skin wound model established in healthy Sprague–Dawley rats. As depicted in Supplementary Figure S5A, the SPBA was topically administered to the 1.5-cm acute wounds. Visual examination of digital wound photographs (Supplementary Figure S5B) and quantitative analysis of the wound-closure curve (Supplementary Figure S5C) demonstrated that the reduction in wound area within the SPB, SPA and SPBA groups was significantly expedited when compared to the control group.

Quantitatively, by the third day following treatment, the average lesion closure rates for the SPB (54.59%), SPA (64.16%) and SPBA (74.36%) groups were considerably elevated relative to the untreated group (19.33%). Although the SP group (30.88%) showed an improvement, it did not reach statistical significance compared to the control. This positive trend continued to Day 7, with closure rates reaching 49.43% (SP), 65.10% (SPB), 73.25% (SPA) and 82.69% (SPBA), all demonstrating superior healing compared to the control (31.54%). By Day 14, the SPBA group achieved near-total wound closure, with an average healing rate of approximately 98.03%, representing the highest among all groups. Although this rate surpassed those of the SP (88.98%), SPA (92.56%) and control (67.23%) groups, no statistically significant disparity was noted upon comparison with the SPA group (95.58%). Collectively, these findings underscore the significant capacity of the SPBA to promote *in vivo* wound regeneration in normal wound.

**Figure 5:**
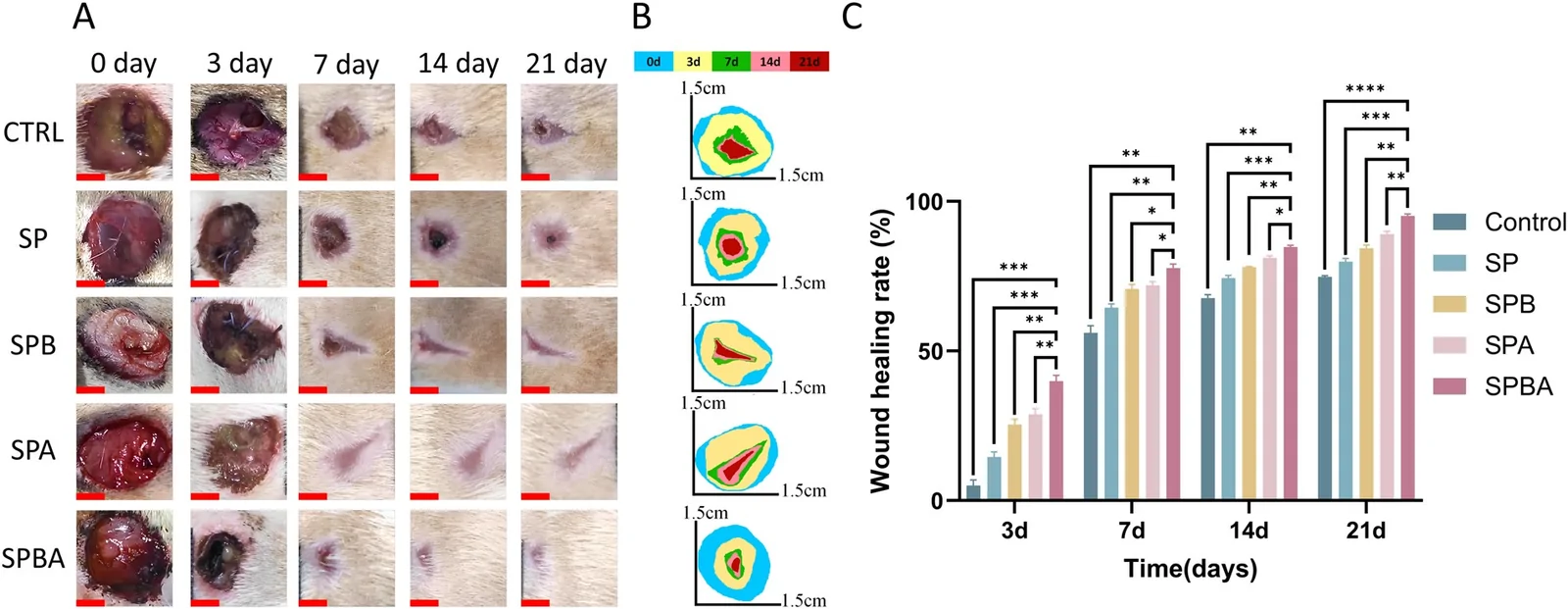
SPBA promotes healing of diabetic wounds. (**A**) Representative images illustrating the recovery process in diabetic models (scale bars: 4.5 mm). (**B**) Schematic representation of wound-healing progression under various treatments over 21 days. (**C**) Quantitative data on relative wound area at distinct time points (*n* = 3) (*P < 0.05, **P < 0.01, ***P < 0.001, ****P < 0.0001).

To further explore the influence of SPBA in accelerating diabetic skin lesion repair, our team established complete-dermal injuries on the dorsal aspect of diabetic SD rats. Figure 5 illustrates that the lesion’s condition was documented through photography at different time points, and the rate of healing was subsequently computed based on lesion size. The results indicated that the wound-healing rate of the SP, SPB, SPA and SPBA groups significantly improved after 3 days of treatment, with wound-closure rates of 14.53 ± 1.71%, 25.35 ± 1.96%, 28.83 ± 1.92% and 39.94 ± 1.92%, respectively. All these values were markedly elevated compared to the control group (5.05 ± 1.86%). Due to prolonged inflammation and an intense inflammatory response, the control group still exhibited a low wound-healing rate at Day 7 (56.02 ± 2.43%), not exceeding 60%. In contrast, the SP (64.51 ± 1.29%), SPB (70.73% ± 1.60%), SPA (71.98% ± 1.23%) and SPBA (77.66% ± 1.42%) groups showed a significant increase in the wound-healing rate by Day 7. With advancing time, the wounds managed with SPBA steadily exhibited the most favorable recovery trajectory across all groups. By the 21st day, the wounds within the SPBA group had achieved nearly full closure, displaying a recovery rate surpassing 95% (95.18 ± 0.66%).

**Figure 6:**
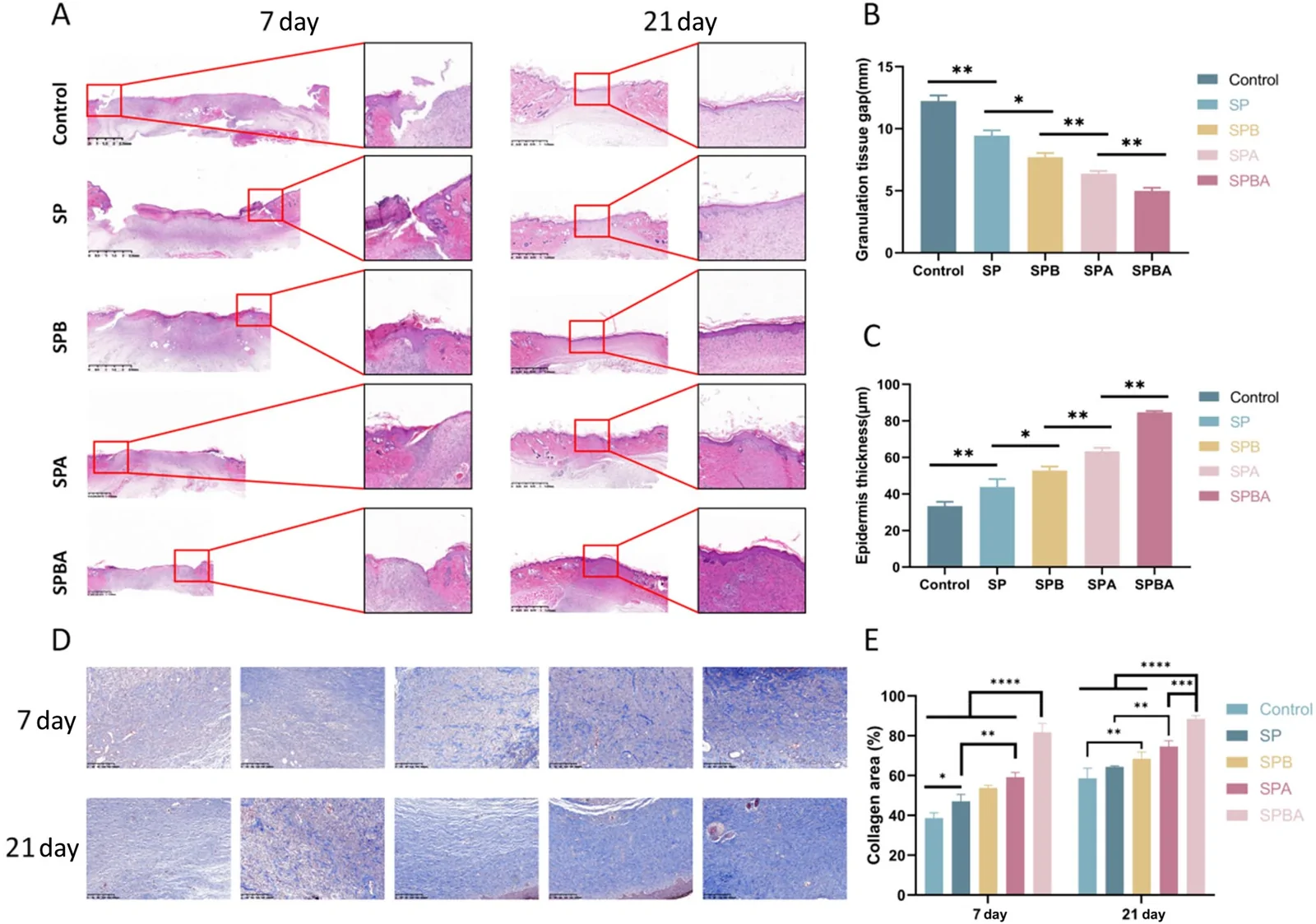
Histological analysis of diabetic wound tissue. (**A**) H&E staining of diabetic wound on Days 7 and 21 (scale bars: 1.25 mm). (**B**) Quantification of the cross-sectional length of different wounds on Day 7 (*n* = 3). (**C**) Quantification of epidermis thickness of various wounds (*n* = 3). (**D**) Masson staining to assess the collagen content in the diabetic wound on Days 7 and 21 (scale bars: 200 μm). (**E**) Quantification of collagen deposition density in different groups (*n* = 3) (*P < 0.05, **P < 0.01, ***P < 0.001, ****P < 0.0001).

Subsequently, quantitative assessment of histological wound cross-sectional dimensions corroborated the aforementioned experimental results. As depicted, HE staining demonstrated notable disparities in lesion size across the groups following 7 days of intervention (Figure 6A and B). The unhealed edge length measured 9.45 ± 0.43 mm for the SP group, 7.71 ± 0.33 mm for its SPB counterpart, 6.38 ± 0.21 mm for the SPA group, while the control group measured 12.24 ± 0.44 mm. The SPBA group exhibited the best wound-healing status, with an unhealed edge length of 5.00 ± 0.24 mm (*P *< 0.01).

Re-epithelialization is regarded as a pivotal stage in wound recovery, establishing an initial protective layer that impedes microbial invasion and mitigates fluid depletion [57]. As shown in Figure 6C, by Day 7, a thin layer of epidermal tissue was visible in the SPBA group’s wounds, while the wounds in the other groups remained open. By Day 21, the SPBA group showed the best wound recovery, with a relatively complete epidermis having a thickness of 84.74 ± 0.72 μm. The control group still exhibited an incomplete epidermis. On Day 7 of wound recovery, the control group had the largest wound diameter (12.24 ± 0.44 mm), while the SP hydrogel significantly promoted wound healing (9.45 ± 0.43 mm). The wound recovery in the SPB (7.71 ± 0.33 mm) and SPA (6.38 ± 0.21 mm) groups was further improved compared to the SP group, confirming the promotional effect of BBR and ASCs on wound healing. The wound healing status of the SPBA group (5.00 ± 0.24 mm) was significantly superior to that of the other groups, exhibiting statistically significant disparities (*P* < 0.01).

Collagen deposition is also crucial for the formation of the ECM [58]. Masson staining results indicated that following 7 days, the collagen fiber density in the SPB group was 2.1 times that of the control group (Figure 6D and E, P < 0.0001), indicating that BBR promotes collagen expression around the wound. The collagen fiber density within the SPBA group was notably greater than that observed in the remaining groups. By Day 21, the collagen fiber density in all groups had substantially risen when contrasted with Day 7, with the SPBA group exhibiting the highest collagen density, which was 1.5 times that of the control group (*P* < 0.0001), which collectively suggests a marked enhancement in ECM deposition within SPBA-treated wounds.

### Immunohistochemistry

**Figure 7:**
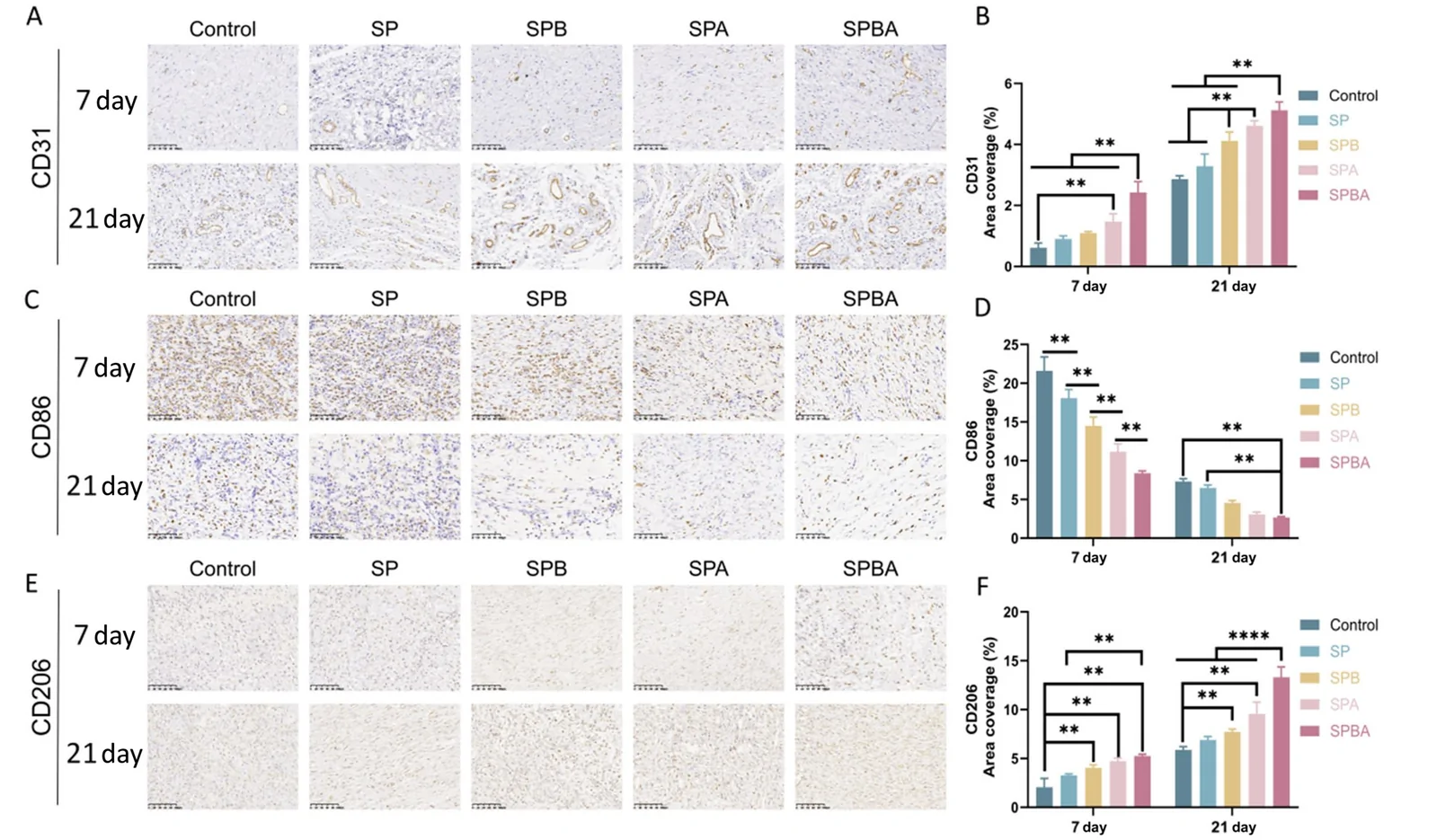
Immunohistochemistry identifies the macrophage transformation and neovascularization. (**A**, **C**, **E**) Immunohistochemical staining with anti-CD31, CD86, and CD206 antibodies on diabetic wound in each group was performed and analyzed on Days 7 and 21 (scale bars: 100 μm). (**B**, **D**, **F**) The quantitative data of the total number of CD31-, CD86- and CD206-positive cells (*n* = 3) (**P < 0.01, ****P < 0.0001).

We utilized CD31 immunohistochemistry to assess angiogenesis in the wounds. Compared to the control group, the expression of CD31 in the SPB, SPA and SPBA groups was significantly elevated, as the results showed (Figure 7A and B). The expression of CD31 in the SPBA group was significantly higher than that observed in the remaining groups, reaching 5.7 times that of the control group (*P* < 0.01), indicating that SPBA significantly promoted *in vivo* angiogenesis.

To investigate the *in vivo* inflammatory regulation of SPBA on diabetic wounds, we performed immunohistochemical staining using anti-CD86 and anti-CD206 antibodies. As shown in Figure 7C–F, on Day 7 after intervention, the expression level of CD86 in the SPBA group was significantly lower than in the other groups (*P *< 0.01). In addition, the expression of the anti-inflammatory M2 macrophage marker was significantly elevated in the SPBA group compared to all the remaining groups (*P *< 0.01). These findings suggest that SPBA exerted a positive anti-inflammatory effect, specifically by promoting the transformation of macrophages from the pro-inflammatory M1 type to the anti-inflammatory M2 type around the wound, thereby reducing inflammatory damage.

After 14 days of wound intervention, the mRNA expression levels within the wound tissue were examined. As shown in Supplementary Figure S6, the expression levels of inflammatory factors including IL-1β, iNOS and TNF-α in the SPBA-treated wound tissue were significantly reduced. In contrast, the expression of anti-inflammatory and pro-healing factors such as IL-10 and TGF-β was significantly increased. Compared to the control group, the SPBA-treated wound tissue showed a marked improvement in the inflammatory response, which was likely due to the regulation of macrophage polarization. This, in turn, led to a significant enhancement in the expression of CD31, VEGF and type I collagen in the wound.

## Conclusion

In this study, we successfully developed an SPBA with adequate mechanical strength and properties conducive to ASC growth by modulating its physicochemical characteristics. The hydrogel’s optimal pore size and stiffness enhanced the activity of encapsulated ASCs, thereby promoting their paracrine functions. Both *in vitro* and *in vivo* experiments demonstrated that SPBA ameliorated the inflammatory response of diabetic wounds by regulating macrophage polarization, which subsequently promoted angiogenesis, collagen deposition and re-epithelialization in the wound. These findings suggest that SPBA exerted a positive anti-inflammatory effect, specifically by promoting macrophage transformation from the pro-inflammatory M1 type to the anti-inflammatory M2 type around the wound, thereby reducing inflammatory damage.

## Supplementary Material

rbag151_Supplementary_Data

## Data Availability

The raw data and processed data required to reproduce these findings are available from the corresponding author upon request.
