# Supplementary material for: Synergistic effects of berberine and adipose stem cells in a silk fibroin-PEG hydrogel for diabetic wound healing via macrophage polarization modulation
Source: Regen Biomater. 2026 Jul 21;13:rbag151. doi: 10.1093/rb/rbag151 (PMC13557628; doi:10.1093/rb/rbag151)
Supplement: Supplementary file 1 [file rbag151_supplementary_data.docx]

*Supporting Information for*

****Synergistic Effects of Berberine and Adipose Stem Cells in a Silk Fibroin-PEG Hydrogel for Diabetic Wound Healing via Macrophage Polarization Modulation****

Xiaohao Hu ^1#^, Jia Xu ^1, 3, 4#^, Yanping Zhong ^4#^, Weihao Zheng ^1, 3^, Chonglin Jiang ^1, 3^, Yuer Zuo ^2^, Ke Ma ^5^, Sheng Xu ^1, 3, 4^*, Li Zheng ^1, 3, 4^*, Hongmian Li ^2^*, Jinming Zhao ^1, 3^

1 Collaborative Innovation Centre of Regenerative Medicine and Medical Bioresource Development and Application Co-constructed by the Province and Ministry, The First Affiliated Hospital of Guangxi Medical University, Nanning, 530021, China.

2 Department of Plastic and Aesthetic Surgery, The People's Hospital of Guangxi Zhuang Autonomous Region & Research Center of Medical Sciences, Guangxi Academy of Medical Sciences, Nanning 530021, China.

3 Guangxi Engineering Center in Biomedical Materials for Tissue and Organ Regeneration, The First Affiliated Hospital of Guangxi Medical University, Nanning, 530021, China.

4 Life Sciences Institute, Guangxi Medical University, Nanning, 530021, China.

5 Department of Plastic & Cosmetic Surgery, The First Affliated Hospital of Guangxi Medical University, Nanning, 530021.China.

*Corresponding author: Email：lihongmian@gxmu.edu.cn (H. Li); xusheng@gxmu.edu.cn (S. Xu); zhengli224@163.com (L. Zheng)

\# The authors contributed equally to this work.

**Figure S1.** CCK8 assay was used to determine the optimal dosage of Berberine.

**Figure S2.** The 3D image of LIVE/DEAD staining of ASCs withinSPB hydrogel.

**Figure S3.** The representative flow cytometry gating strategy for macrophage populations (M1 = CD86+ and CD11b+; M2 = CD206+ and CD11b+).

**Figure S4.** Respective Elisa standard curves.

**Figure S5.** SPBA promotes healing of diabetic wounds.

(A) Representative images of recovery process in normal wound. (B) Schematic diagram of the wound healed by different treatments during 14 days. (C) Quantitative data of relative wound area at different time points.(^*^P<0.05, ^**^P<0.01, ^***^P<0.001, ^****^P<0.0001, P<0.05)

**Figure S6.** The mRNA expression of cytokines in the wound following different treatments. (^*^P < 0.05, ^**^P < 0.01)

| **Gene Name** | **Primer sequence** |
| --- | --- |
| **CD31** | **GTCATTGCCGCCTTGATAGTTG** |
|  | **GGAGTCGTAATGGCTGTTGGTT** |
| **VEGF** | **CGAGGAAAGGGAAAGGGTC** |
|  | **TTCTCCGCTCTGAACAAGG** |
| **IL-1β** | **CTTCAAATCTCACAGCAGCAT** |
|  | **CAGGTCGTCATCATCCCAC** |
| **iNOS** | **TTGGAGCGAGTTGTGGATTGTT** |
|  | **GGAAGCCTCTTGTCTTTGACCC** |
| **TNF-α** | **AGCATGATCCGAGATGTGGAA** |
|  | **TAGACAGAAGAGCGTGGTGGC** |
| **TGF-β1** | **AACAATTCCTGGCGTTACCTT** |
|  | **GCCCTGTATTCCGTCTCCTT** |
| **IL-10** | **TGCTATGTTGCCTGCTCTT** |
|  | **GTCTGGCTGACTGGGAAGT** |
| **Collagen I** | **TGGCAACCTCAAGAAGTCCC** |
|  | **CCATCCACAAGCGTGCTGTA** |
| **β-Actin** | **TGACGTTGACATCCGTAAAGACC** |
|  | **GTGCTAGGAGCCAGGGCAGTAA** |

**Table S1.** Primer sequences used for qRT-PCR
